# Association of heteromorphism of chromosome 9 and recurrent abortion (ultrasound diagnosed blighted ovum): A case report

**Published:** 2014-05

**Authors:** Fatemeh Baghbani, Salmeh Mirzaee, Mohammad Hassanzadeh-Nazarabadi

**Affiliations:** *Department of Medical Genetics, Faculty of Medicine, Pardis Campus, Mashhad, Iran.*

**Keywords:** *Recurrent abortion*, *Heteromorphism*, *Chromosome no. 9*, *Pregnancy complication*, *Blighted ovum*

## Abstract

**Background:** Chromosomal disorders are the most common cause of first trimester spontaneous abortion. Among the human chromosomes, chromosome no.9 was the most common structural chromosomal variant and it is not thought to be of any functional importance, which often considers as a normal variation in structural polymorphisms, nevertheless there are some studies which claim that there is an association between heteromorphism of chromosome no.9 and some pregnancy complication.

**Case: **To postulate any correlation between chromosome no. 9 heteromorphism and recurrent abortion, chromosomal analysis was performed on the basis of G-banding technique at high resolution for a couple with the history of 4 ultrasound diagnosed blighted ovum and Chromosome constitution appeared with chromosome no.9 heteromorphism in all 30 metaphases screened for both partners (9p11-q13).

**Conclusion:** Observation of reproductive failure in couples with heteromorohic pattern of chromosome no.9 suggests that, although the heteromorphism of chromosome no.9 is not a rare condition which often consider as a normal variation with no evidence of any phenotypic effect of patient, nevertheless it seems as if the location of heteromorphic region maybe interfere with meiotic events like the phenomenon of crossing over or miotic segregation of fertilized egg that eventually lead to the development of fertilized eggs with chromosomal abnormalities leading to the possibility of anemberyonic pregnancy, therefore chromosomal analysis for detecting of chromosome no.9 heteromorphism for couples with the history of ultrasound diagnosed blighted ovum will be strongly suggested.

## Introduction

Blighted ovum, also known as anembryonic pregnancy, may lead to cause of early miscarriage at very early in the first trimester of pregnancy (1, 2). The main characterization of an anembryonic gestation is a normal-appearing gestational sac, but the absence of an embryo (1). This is most likely to happen as a result of early embryonic death with continued development of the trophoblast (2). Three main reasons in relation to blighted ovum might be suspected; chromosomal abnormality, abnormal cell division, as well as poor quality of the sperm or the egg which might be resulted in chromosomal abnormalities leading to the cause of blighted ovum (3, 4). It has been reported that; One third of the products of conception from spontaneous abortion occurring at or before 8 weeks of gestation are blighted ovum (5, 6). 

Overall, chromosomal aberrations are the cause of 50% of first trimester spontaneous abortions (7). The most frequent structural chromosome abnormalities in recurrent miscarriage are translocations (reciprocal translocation (62%), robertsonian translocation (16%), inversions (16%) and deletions and duplications (3%) (8). Among the human chromosomes, chromosome no. 9 occurs as a common structural variant which are commonly found in cytogenetic reports and can be observed in 6-8% in the general population (5, 9). 

There are some evidences which claim that, there might be potential adverse effects of heteromorphic morphology of chromosome no. 9 and reproductive ability. Until now, the role of any clinical significance of such structural heteromorphism or polymorphism or what is known as heteromorphism which is not thought to has any functional importance is not well understood, but there are some evidences which claim that, there might be a potential adverse effects of heteromorphic morphology of chromosome no.9 and reproductive ability (8).

## Case report

**Figure 1:**
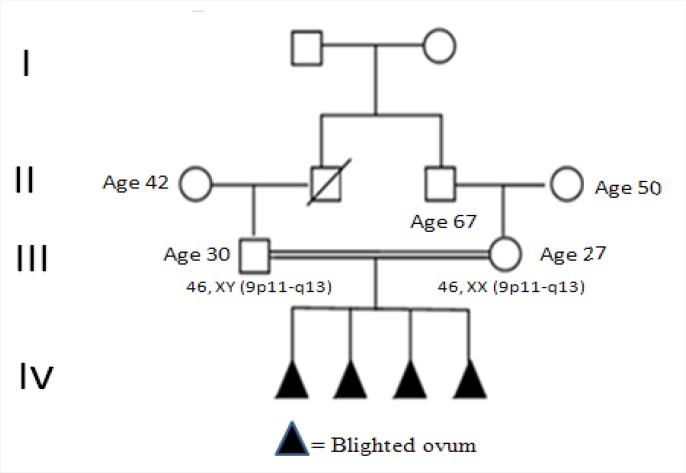
Family pedigree with heteromorphism of Chromosome 9

In this study we report a history of habitual miscarriages of a couple with unknown etiology. The 30-year-old man and 27-year-old woman who had consanguineous marriage with no history of normal gestation and have had four recurrent spontaneous abortions which diagnosed blighted ovum at 8-10 weeks of gestation. The family pedigree is shown in (Figure 1). 

**Figure 2:**
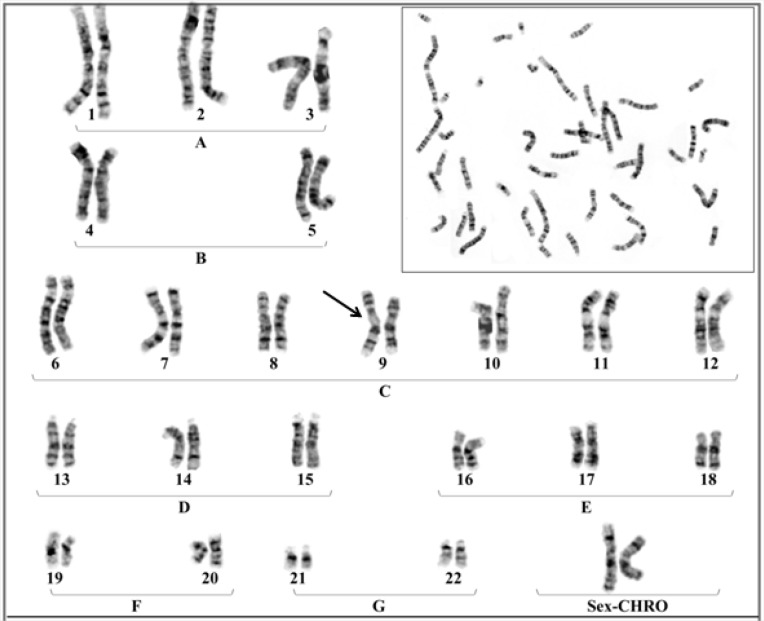
Chromosomal constitution; 46, XX (9p11-q13)

**Figure 3:**
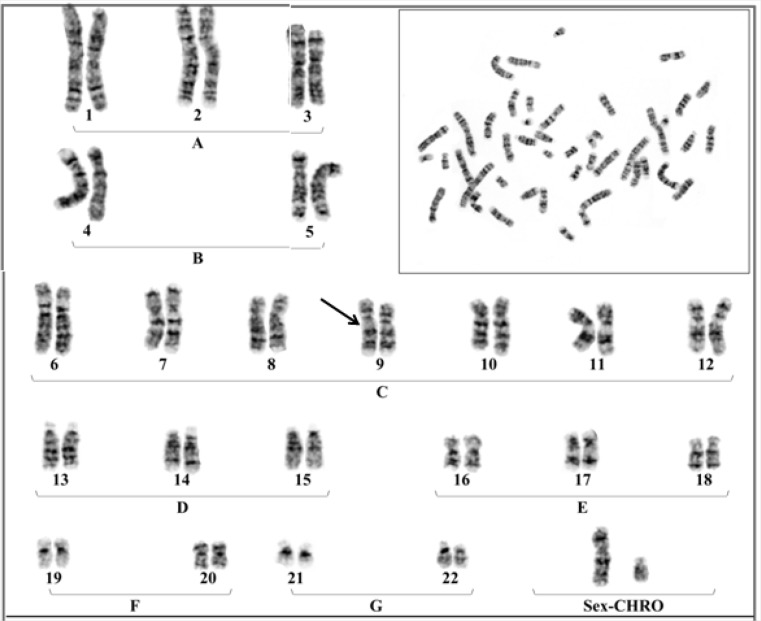
Chromosomal constitution; 46, XY (9p11-q13)

They were initially referred to the infertility center for searching the cause of such pregnancy complication. Hormonal, immunological and anatomical factors of uterus were normal and the partner particularly mother had no underlying disease related to such complication. After obtaining informed consent, chromosomal investigation was performed on the basis of G-banding technique at high resolution. The results showed similar heteromorphism of chromosome no.9 at the same location in both partner [46, XX (9p11-q13) and 46, XY (9p11-q13)] with normal phenotypes (Figure 2, 3). 

## Discussion

Fetal chromosomal abnormalities are the most common case of spontaneous abortion. According to the recent studies chromosomal abnormalities are responsible for 50% of the first trimester spontaneous miscarriage; most of them about 86% are numerical whereas structural and other unknown genetic disorders are 6% and 8% respectively (7). However the most common structural abnormality in couple with experience of recurrent abortion is balanced rearrangement (10). A blighted ovum is characterized through of ultrasound examination by the absence of embryo in the gestation sac (an embryonic pregnancy). One-third of the products of conception from spontaneous abortion occurring at or before 8 weeks of gestation are blighted ovum or an embryonic pregnancy (6).

Most chromosomal abnormalities in the embryo occur de novo. Rarely these defects are inherited as a consequence of parental karyotype such as balanced translocations. Recent study claimed that, the most common structural abnormalities in couple with experiences of recurrent abortion are; reciprocal translocation (62%), robertsonian translocation (16%) whereas pericentric and paracentric inversion are 16% and 3% for other structural chromosome abnormalities   (11). Among the human chromosome, chromosome no.9 appears with the high frequency of structural heteromorphism, which is a natural variation that occur 1-2% among the individuals in the general population and transmitted through family as mendelian trait (8).

Molecular studies suggested that the structural organization of chromosome no.9 makes it apparently prone to breakage and may be associated with a higher incidence of observed aberrant morphology of this chromosome (12, 13). The risk of miscarriage in the carriers depends on the affected chromosomal region (14). Since chromosomal heteromorphism is considered as balanced chromosomal rearrangements and may not cause clinical feature in carriers, unless polymorphic region has adverse effect on miotic events or chromosome breakpoint occurs within specific regulatory or structural region of gene which affects gene expression, however other studies claimed that there is an association dose exist between structural morphology and some clinical phenotypes (10). 

Therefore parental chromosomal analysis is important for appropriate genetic counseling in relation to an embryonic pregnancy (15, 20). Although several authors showed no any evidence between parental chromosome rearrangements as risk factor of blighted ovum, nevertheless a number of recent studies, focused on the increased prevalence of some structural abnormalities of chromosomes in couple with history of an embryonic pregnancy in comparison with the general population (18, 21). Based on the study observations, it seems that the location of heteromorphic region may be interfere with such meiotic events like the phenomenon of crossing over or miotic segregation of fertilized egg that finally leads to development of fertilized eggs with chromosomal abnormalities and produce abnormal embryo (anembryonic pregnancy). 

## Conclusion

In conclusion further investigation will be suggested in couples with the history of blighted ovum pregnancy and chromosome no.9 heteromorphic in order to postulate a correlation between heteromomorphism (chromosome no. 9) and an embryonic pregnancy. 
